# The Effect of a Bidirectional Exchange on Faculty and Institutional Development in a Global Health Collaboration

**DOI:** 10.1371/journal.pone.0119798

**Published:** 2015-03-23

**Authors:** Benjamin E. Bodnar, Cassidy W. Claassen, Julie Solomon, Harriet Mayanja-Kizza, Asghar Rastegar

**Affiliations:** 1 Division of General Internal Medicine and Primary Care at Brigham and Women’s Hospital, Boston, Massachusetts, United States of America; 2 Medicine at Harvard Medical School, Boston, Massachusetts, United States of America; 3 District Clinical Advisor with Partners in Health, Butaro, Rwanda; 4 University of Maryland Medical Center, Baltimore, Maryland, United States of America; 5 J. Solomon Consulting, LLC, Mountain View, California, United States of America; 6 Department of Medicine, College of Health Sciences, Makerere University, Kampala, Uganda; 7 Department of Medicine, Yale School of Medicine, New Haven, Connecticut, United States of America; World Health Organization, SWITZERLAND

## Abstract

**Purpose:**

The MUYU Collaboration is a partnership between Mulago Hospital-Makerere University College of Health Sciences (M-MakCHS), in Kampala, Uganda, and the Yale University School of Medicine. The program allows Ugandan junior faculty to receive up to 1 year of subspecialty training within the Yale hospital system. The authors performed a qualitative study to assess the effects of this program on participants, as well as on M-MakCHS as an institution.

**Methods:**

Data was collected via semi-structured interviews with exchange participants. Eight participants (67% of those eligible as of 4/2012) completed interviews. Study authors performed data analysis using standard qualitative data analysis techniques.

**Results:**

Analysis revealed themes addressing the benefits, difficulties, and opportunities for improvement of the program. Interviewees described the main benefit of the program as its effect on their fund of knowledge. Participants also described positive effects on their clinical practice and on medical education at M-MakCHS. Most respondents cited financial issues as the primary difficulty of participation. Post-participation difficulties included resource limitations and confronting longstanding institutional and cultural habits. Suggestions for programmatic improvement included expansion of the program, ensuring appropriate management of pre-departure expectations, and refinement of program mentoring structures. Participants also voiced interest in expanding post-exchange programming to ensure both the use of and the maintenance of new capacity.

**Conclusions:**

The MUYU Collaboration has benefitted both program participants and M-MakCHS, though these benefits remain difficult to quantify. This study supports the assertion that resource-poor to resource-rich exchanges have the potential to provide significant benefits to the resource-poor partner.

## Introduction

Interest in global health programming at academic medical institutions has grown significantly in recent years [1–4]. Most collaborations are built around international health electives (IHE), during which students or residents from the resource-rich partner institution spend time observing, working and learning on-site at the resource-poor partner institution [1, 3, 5–9]. Bidirectional exchanges, in which students, residents or other faculty from the resource-poor partner spend time within the resource-rich environment, are a relative rarity [5, 10]. This can be attributed, in large part, to financial issues, but may also relate to historical concerns about the applicability of training undertaken in a resource-rich environment when practicing in a resource-poor environment, the fear of brain drain, and limitations on visiting physicians’ ability to participate in clinical training [11]. Inasmuch as resource-poor to resource-rich partner exchanges remain rare, there have been no publications detailing the effects of a bidirectional exchange on either the resource-poor partner participants or on the resource-poor partner institution on the whole.

The Department of Internal Medicine at the Yale School of Medicine maintains one of the oldest global health programs in the country, with regular IHE programming in place for residents since 1981 [6]. As a part of this program, the Makerere University-Yale University Collaboration (MUYU) was established in 2006 with Mulago Hospital and the Makerere College of Health Sciences (M-MakCHS) in Kampala, Uganda. Mulago Hospital is a national referral hospital operated by the Ugandan Ministry of Health. It is Uganda’s largest public hospital, with roughly 1500 beds. Patient volume is very high, with the total census often exceeding the hospital’s stated capacity. Resources are limited and shortages of pharmaceuticals, staff, and other supplies are frequent. Mulago is also a teaching hospital, serving as the main clinical teaching site for medical students from MakCHS, and hosting residency programs in a number of specialties.

Since 2007, the MUYU Collaboration has included a resource-poor to resource-rich partner exchange in which M-MakCHS staff are sponsored to train at Yale-New Haven Hospital (YNHH) and its affiliates. With the exception of the earliest years of the exchange (which included Ugandan senior residents), the resource-poor partner participants are junior faculty at M-MakCHS, who have completed their specialty internal medicine training and attained a faculty position at the institution. Individual participants are initially selected by the departmental leadership at M-MakCHS and then interviewed in person by a Yale faculty member to ensure that the candidate is aware of his or her role at Yale, and is committed to continuing his or her work in Uganda upon completion of the rotation. The participating faculty are asked to choose a subspecialty area focusing on non-communicable diseases, spend a minimum of a year in that subspecialty at M-MakCHS, and are then sponsored for further training at YNHH and it affiliates under the supervision of a Yale mentor in this field. Early in the exchange, visits were sometimes as short as three to six months; however they now generally last one year, which allows the participating Ugandan faculty to be recognized as subspecialists upon their return to Uganda. Increasingly, participants are encouraged to design their own program with input from their mentor at Yale. Funding is provided by a combination of grants from the Yale School of Medicine, The Yale-Mulago Fellowship Foundation, Johnson & Johnson Corporate Contributions (the charitable arm of the Johnson & Johnson Corporation), as well as other groups (such as the International Society of Nephrology). The program does not have formal follow-up or alumni activities, although many participants maintain active relationships with their mentors or the MUYU program after completing their subspecialty training. As of the time of this study, the exchange had enabled a total of 12 Ugandan physicians to complete training at Yale. The MUYU Collaboration also supports an ongoing Yale to M-MakCHS exchange, in which medical and physician assistant students, residents, and attending faculty spend an average of six weeks at Mulago Hospital.

We carried out a study to assess the effects of training Ugandan M-MakCHS faculty at Yale, including the impact of this training on the individual physician, as well as the effect of this training and the MUYU Collaboration on M-MakCHS as an institution.

## Methods

Initial data was collected between November 2011 and February 2012 via a confidential online survey completed by Ugandan MUYU participants. The survey captured demographic information and basic information on participants’ experience with the program. This data served as the basis for constructing the semi-structured interview protocol used in the qualitative study discussed below.

The interview protocol was designed with input from project team members at both Yale and M-MakCHS, and was intended to elicit information on the specific effects, challenges and needs of the MUYU exchange program from M-MakCHS faculty participants. The protocol included questions on the exchange experience; the impact of the exchange on participants’ knowledge, clinical, and teaching skills, as well as their attitudes and professionalism; and the effect of the exchange on M-MakCHS as an institution. Interviewees were also asked to discuss areas in which further capacity building is needed at M-MakCHS, and to address the potential contribution of the program to brain drain. Specific suggestions for improving the MUYU Collaboration were also elicited. (See S1 Text. Qualitative Interview Protocol of M-MakCHS Faculty MUYU Participants.)

The interviews were carried out in person from April to October of 2012 by one female Ugandan interviewer with no prior connection to the MUYU program. The interviewer was primarily employed as a Lecturer in Disease Control and Environmental Health at Makerere University School of Public Health, and had previous experience in qualitative interviewing. As the Makerere University School of Public Health shares a campus with Mulago hospital, the interviewer was known to a small number of the interviewees, but had no significant relationship with any of them. Members of the research team (C.C. and J.S.) trained the interviewer on the interview protocol. The interviewer was permitted to add probes and ask clarification questions, as well as to skip specific questions if the topic had already been discussed by the time the question was reached in the protocol. Interviewees, for their part, were informed that they could skip questions that they did not wish to answer. Only one of eight interviewees elected to skip portions of the interview. All interviews were conducted in English, which is the primary language of Ugandan academic institutions, and in which all interviewees were fluent. Only the interviewer and interviewee were present at interviews, which generally took place in a conference room or other private area of the Mulago Hospital campus. Each participant was interviewed once.

The length of the interviews ranged from 33 to 84 minutes. The interviewer audio-recorded the interviews and also typed up field notes, immediately following each interview, concerning key themes expressed by the interviewee and observations about the interviewee’s apparent level of comfort and focus. The interviewer also noted any other factors that may have influenced the content or framing of the discussion. The field notes were reviewed and incorporated into later thematic analysis.

The interviews were transcribed verbatim by a professional transcription service. Interviews were designed not to include overt identification of participants, and files and transcriptions were processed without identifying demographic information. Transcriptions were not shared outside the research team. The transcriptions were entered into Dedoose (www.dedoose.com), a web-based, cross-platform application for analyzing qualitative and mixed-methods research data. Dedoose was used for all of the project’s coding and querying.

Based on the interview template, a preliminary codebook was designed which included both topical codes (i.e., codes referring to specific interview questions) and thematic codes (i.e., codes reflecting the underlying message or content of the excerpt, such as “Expected Benefits,” “Actual Benefits,” “Expected Costs,” “Actual Costs,”). One of the authors (B.B.) developed the initial draft of the codebook, and another team member (J.S.) provided feedback. The codebook was further revised concurrently with coding of the initial three interviews, and then all interviews were (re)coded with the updated scheme.

Under the final coding scheme, author B.B. coded all interviews, after which author J.S. reviewed a subset of the codings. Several minor questions about the coding arose in the review process. The two team members discussed and resolved these questions before a review and revision of the relevant coding was carried out. Although all interviews were fully coded, saturation was reached after three-quarters (6 of 8) of the interviews had been coded, with few new ideas or themes presenting themselves in the final interviews.

The analysis used an inductive approach, based on grounded theory [12, 13], in which author B.B. ran analytic queries to retrieve code information by topical and thematic category and at the intersection of multiple categories. The outputs of the queries were then used to develop thematic memos, with illustrative examples, focused on the main interview topics, as discussed above. The various thematic memos were then synthesized into key findings, which were reviewed by all authors.

### Ethics Statement

Participants provided informed consent prior to participation in both the online survey and interview portions of the study (in electronic and written form, respectively). The study was approved by the Institutional Review Board (IRB) of Makerere University College of Health Sciences and deemed exempt by the Yale University School of Medicine IRB.

## Results

### Participants

All twelve individuals who had completed their participation in the MUYU exchange at the time of the study were invited to be interviewed. Invitations were sent by email, and subsequently by phone, if needed. Eight participants (67% of eligible respondents) completed interviews. The remaining participants declined to be interviewed due to the time requirements of participation, or were unable to be interviewed due to scheduling conflicts. Those interviewed included 5 respondents who had participated in the exchange as junior faculty, and 3 respondents who had participated during their residency—two of whom became junior faculty at M-MakCHS upon their return, and one of whom is now a faculty member at another teaching hospital in Uganda. Respondents were between the ages of 34 and 39 (mean 36.5). Seven of the eight respondents were male (9 of the 12 eligible participants were male). All respondents had attended medical school at Makerere College of Health Sciences. Only a minority (3 of 8) of respondents had completed internship at Mulago Hospital, although all had completed their residency training at Mulago. A majority of interviewees (5 of 8) had participated in some medical training abroad prior to MUYU participation, of whom many (3 of 5) had spent time at McMaster University in Ontario, Canada, while the other 2 participants had spent time at other US institutions. Prior international experiences were generally significantly shorter than MUYU exchange participation, though they ranged widely in duration, most often lasting from several weeks to several months. During their MUYU exchange, interviewees spent a mean of 7.6 months working at Yale-affiliated hospitals (range 3 to 12 months, with the shortest exchanges occurring in the program’s first year, 2007). At the time of interview, a mean time of 5.1 years (range 2 to 9) had elapsed since completion of residency training, and 3.3 years (range 2 to 5) had elapsed since MUYU exchange participation. Two of the respondents trained in cardiology, two in nephrology, one in hematology/oncology, one in rheumatology, one in gastroenterology, and one in general medicine.

### Findings

Analysis of interview data revealed themes that have been grouped under four topics: 1) programmatic benefits and impact, 2) barriers and difficulties, 3) the effects of the program on brain drain, and 4) suggestions for programmatic improvement.

### Programmatic Benefits and Impact

#### Effect on knowledge

There was universal agreement amongst interviewees that the greatest impact of the MUYU program was on the fund of general and specialized medical knowledge of participants. Half of respondents specifically mentioned improved ability to interpret medical literature or practice evidence-based medicine (EBM), and several mentioned improved ability to read and interpret various imaging modalities. Interviewees overall expressed great satisfaction with the knowledge they gained. For example, one participant stated:

“…I learned how to do dialysis—peritoneal dialysis from there [Yale]. I learned how to manage patients with peritoneal dialysis from there. Management of patients with acute kidney [injury]– got it from there. Interpretation of results like radiology co-investigations, ultrasounds, CT scans, and all of that. Also the methodology of teaching… even professionalism in patient care – all those things I learned from Yale and I’m practicing them.” *Interviewee number withheld for confidentiality*

#### Effect on clinical practice

All respondents indicated that the exchange had a considerable effect on their role and practice as a clinician. Half noted that an increased use of EBM had affected their clinical practice. One interviewee noted:

“I still do read a lot of journal articles, and I do fortunately now understand and interpret them accordingly. I put some of the clinical information accrued from these journals to real practice… if the evidence is sound, it is worth practicing it.” *Interviewee 4*

Most respondents also described a significant effect on their doctor-patient and doctor-family relationships as a result of the exchange, which they characterized as including increased communication, and increased valuation of patient autonomy and independence. One interviewee described his post-exchange practice as follows:

“I give patients time. … I take time to speak to the patient about the disease. Maybe not so much how the disease comes but how we are going to manage and what options we have available. But I also tell them what options they would get if they went elsewhere… And I found that it gives the patients—they feel a lot of relief and they've been respected.” *Interviewee 3*

#### Effect on medical education

All interview respondents cited some positive effects of the exchange on medical education at M-MakCHS, generally via its effect on their skills as teachers. Most interviewees referred to an increase in their own knowledge base, while several also mentioned changes to their teaching methods (such as increased teaching of EBM and use of journal articles as teaching sources). Several also described alterations to the attending-student relationship, leading to a more Socratic, less didactic approach to teaching. One interviewee noted:

“A good teacher should be very knowledgeable. The knowledge I acquired has helped me to be able to speak confidently learning to question the evidence… As a teacher I know that I have a good knowledge base. But also being compassionate. I’m no longer your boss when I’m on the ward. I take all the ideas from the students and then we discuss them.” *Interviewee 1*

Interviewees also referred to the recent establishment of an office of resident medical education and a chief resident position at Mulago. These innovations were the result of a project created by a MUYU alumnus, with financial and logistical support from the MUYU program. These developments were felt to reflect an increased interest in education within the department of medicine as a whole, and to have been beneficial to resident education.

#### Effect on research

MUYU participants reported mixed effects of the exchange on their roles as researchers. Some interviewees noted no significant effect of the exchange on their research careers, though others credited the exchange with increasing their publications (via Yale collaborations), creating mentor-mentee relationships leading to current research, and providing the knowledge base that allowed current research pursuits to progress. Amongst interviewees, there was general agreement that the exchange was not structured primarily to support research skills or projects, and a few suggested that more specific programming should be incorporated to this end.

#### Effect on leadership

Interview respondents reported that the program did not have a significant direct effect on their skills as leaders, although several noted that their leadership roles increased as an effect of program participation. Interviewees explained that promotion to positions of departmental leadership or oversight, as well as informal leadership in the larger Ugandan medical community, were the result of the sought-after specialty skills that they had acquired through the MUYU program.

#### Applicability of skills

While interviewees were not directly asked to comment on the applicability of training in a resource-rich environment upon return to a resource-poor environment, they often commented on this issue. Although some interviewees commented that not all of the knowledge they had gained was immediately applicable in Uganda, none felt that the time spent attaining the knowledge and skills was wasted. Rather, several interviewees specifically noted that the knowledge they gained anticipated technology that soon became available in Uganda, making their knowledge especially valuable. For example, one interviewee noted:

“As you know, they have opened a [cardiac] catheterization laboratory [at Mulago Hospital] … [While at Yale] I managed to see how people do catheterizations and now they’re being done here. So things are, like I told you, two years ago, three years ago, they didn’t look like they would be useful, but right now you can see that the training was really appropriate.” *Interviewee 2*

Other interviewees noted that the knowledge of what is unavailable at M-MakCHS is key for motivating improvements in the medical system, regardless of whether the capacity currently exists for the implementation of these improvements.

#### Effects on professional interpersonal relationships

Interviewees were asked to compare the character of staff relationships (e.g., between physicians and students, residents, or nurses) at Yale and M-MakCHS, as well as comment on any effect that the exchange may have had on those relationships at M-MakCHS. The most notable differences were identified in physician-medical student and physician-nurse relations. Both relationships were routinely characterized as being more distant and involving less bidirectional flow of information at M-MakCHS compared to Yale. Interviewees often described a “gap” between physician teacher and medical student at M-MakCHS, and interactions that were confrontational and tended to use fear as a significant motivator. Most interviewees felt that the closer physician-student relationship at Yale improved teaching productivity relative to M-MakCHS. In contrast, one respondent felt that a kinder, less confrontational relationship led to less motivation amongst the students, and thereby less uniform achievement of expectations. Most interview respondents described a significant change in their relationships with students after exchange participation, which was usually characterized by increased rapport and communication. One stated:

“[At Mulago] I don’t know whether it’s a combination of respect and fear but the professor I mean is held in awe… But that is changing with our program… [The faculty] have actually greatly changed. Now they allow the students to email them, they can even phone them and ask them for a session. Probably that was not there before but after these interventions things have changed a little bit.” *Interviewee 1*

Physician-nurse relations at M-MakCHS were uniformly described as problematic, with no routine communication other than via physician orders, and no structured mechanisms for feedback. Interviewees acknowledged the benefit of more open communication, and a more bidirectional flow of information, but also noted contextual factors limiting the improvement of physician-nurse relations, including the severe nursing shortage in Uganda, and a relatively greater knowledge gap between the physician and nursing groups, which most attributed to the different educational requirements for nurses within the Ugandan and US systems. While a few interviewees did not see a clear effect of the MUYU exchange on their relationship with nurses, the majority of those who commented on the issue described improvements, including increased respect for nurses, increased appreciation of nursing contributions, and increased desire for communication. One interviewee described the changes as follows:

“My relationship with nurses is—I revere nurses a lot more now. I feel that they are more a part of the team than I actually thought they were. I know now that the nurses play and should play and should be allowed to play a much bigger role than we allow them to play in Uganda.” *Interviewee 4*

#### Institutional effects of the Yale to M-MakCHS exchange

As the MUYU Collaboration involves a bidirectional exchange, interviewees were asked to compare the impacts of the Yale to M-MakCHS and the M-MakCHS to Yale exchanges on M-MakCHS as an institution. All interviewees cited some beneficial effects of the Yale to Mulago exchange, though they were mostly described as modest in scale, with some potential to have neutral or even negative effects, depending on the circumstances. The most commonly cited benefit of the Yale to M-MakCHS exchange was the visiting residents’ and faculty’s role in providing on-site teaching, and several noted that visiting residents provided good modeling of patient advocacy skills. A few respondents noted that this exchange also had some positive effect on supporting research collaborations between faculty at the two institutions. The effect of visiting medical students was felt to be relatively neutral: while their insightful questions may benefit ward-based clinical teaching, on balance it was felt that “they learn more than what they bring” *(Interviewee 1)*. The potentially negative effects of the Yale to M-MakCHS exchange were mentioned only abstractly. One respondent noted that the ability of visiting residents and students to adapt to the novel environment is highly variable; while most visitors maintain a positive, flexible attitude and are therefore successful, those individuals who have more difficulty adjusting to the novel environment may become withdrawn, negative or condescending, which may have adverse effects on interpersonal and team dynamics.

#### Institutional effects of the M-MakCHS to Yale exchange

There was widespread agreement that the ongoing M-MakCHS to Yale exchange has had positive effects at a wider departmental, if not institutional level. The exchange was seen to have beneficial effects on patient care, student and resident education, staff relations, and potentially research, mostly via the alterations to individual knowledge, skills, and practices that are described above. There was considerable variation, however, in the interviewees’ assessments of the degree of this larger effect. Amongst respondents who provided specific estimates, two-thirds characterized the effects of the exchange as moderate to large, while the remaining third characterized the effects as small or minimal. Those who described more modest effects of the exchange routinely described the main barriers to achieving a more significant effect as either 1) the small total number of MUYU participants in the wider faculty pool (i.e., the program had not yet reached “critical mass”), or 2) the systems and resource limitations that have been unaffected by the collaboration. Only one respondent expressed pessimism about the capability of the program to have more widespread effects, which he attributed to perceived insurmountable financial and systems issues at play in the larger medical system. The remainder, including those who felt that the effects of the exchange were modest to date, encouraged continuation, if not expansion of the program, to allow benefits to continue to accrue. One interviewee described the slowly accumulating effects of the program as follows:

“Initially it was hard to see the outcomes of this collaboration because we are few. You could not easily tell from the whole pool of many people around our influence—the influence of how Yale has impacted on us. But as we get more and more people from here going to Yale and coming back to practice here… everything is adapting to that American way of doing things, Yale standards.” *Interviewee 7*

### Barriers and Difficulties

The major program-related challenges described by MUYU participants include 1) barriers to participation and learning (such as financial and institutional barriers), and 2) barriers to the application of new skills (such as limited medical resources and systems challenges).

#### Barriers to participation—Financial

Although the MUYU program sponsors travel to and from Yale, housing, and provides a small stipend to support basic necessities, all participants stated that making ends meet was difficult. For many, participation in the program required significant financial sacrifice. The most significant financial concern for most participants was not related to personal expenses, but to the ongoing support of both immediate and extended family, which is common in Ugandan culture. While participants who already held junior faculty positions at M-MakCHS continued to receive their base salary, these participants often still complained of loss of income, as it is common for a significant proportion, if not a majority, of a physician’s gross income to come from private clinical work or other outside activities. The two interviewees who provided numerical estimates noted that their family’s income during participation became roughly one-quarter (20–30%) of what it was prior to participation. All respondents suggested increasing the amount of the stipend or offered other suggestions on methods to decrease the financial burden of participation, such as cost-sharing amongst alumni to support current participants, or pursuing additional sources of support outside the program.

#### Barriers to participation—Licensing, procedural and practical clinical limitations

Due to US licensing and insurance issues, Ugandan participants are generally not permitted to perform any patient procedures and some hands-on examinations; they act as observers under most circumstances. Although interviewees routinely expressed overall satisfaction with their educational achievements during the exchange, many also noted that the inability to perform hands-on clinical procedures was a limiting factor. A few interviewees stated that due to the close, trusting relationship they developed with their mentors or supervisors, they were still able to participate in some procedures. Another noted that although opportunities were not available to perform a certain procedure at Yale (kidney biopsy), it was possible to arrange hands-on supervision for this procedure upon returning to Uganda, and thereby incorporate the procedure into regular practice. Nonetheless, this restriction remained significant for many participants.

#### Barriers to participation—Institutional attitudes regarding participation.

Some participants described initially skeptical institutional attitudes at M-MakCHS regarding participation in the exchange. One early participant summarized his experience as follows:

“The administration [was] usually skeptical. They say: are you going to learn very much from Yale? Are you sure you want to go to Yale? Why don’t you go to India and all those things because of the limitations of the hands-on, but of course when you come back they begin to appreciate the knowledge you have and every day I get five consults from the different departments because they know I’m the expert in the field.” *Interviewee 1*

These attitudes among the administration appear to have changed considerably over time, however, as earlier participants were more likely to describe resistance, whereas later participants described strong support from the administration.

#### Barriers to the application of new skills—Resource limitation after participation

Interviewees universally cited resource limitations after their return to Uganda as a significant barrier to the application of new skills and to programmatic success. The most commonly cited resource limitation was a lack of both durable and disposable medical equipment necessary for clinical care. Interview respondents discussed limited or absent equipment to support diagnostic procedures (such as kidney biopsy or specific laboratory testing), diagnostic imaging modalities, dialysis, surgical capabilities, and other basic patient care requirements. Although interviewees referred to some helpful contributions that had been made by the MUYU program (such as an ultrasound for echocardiogram, and CPAP machines), there was also agreement that, given their modest scale, these donations had little effect on the overall resource limitation. Other resource limitations that were discussed included staffing limitations, both amongst physicians (limiting interviewees’ ability to pursue additional projects) and nurses (limiting interviewees’ ability to foster an improved physician-nurse relationship), as well as a lack of intellectual resources (such as a relatively limited medical library, and access to online medical information content), and technological resources (such as computers and handheld devices). Respondents also cited resource limitations in larger local and national systems as significant problems. Interviewees described barriers including poorly functioning governmental and institutional bureaucracies, lack of easily available financial support for research in general, and non-communicable diseases specifically, and even lack of national infrastructure, such as reliable roads and transportation systems, to allow consistent access to care.

#### Barriers to the application of new skills—Cultural barriers to change

Although the increasing pool of MUYU participants has provided an impetus for increased institutional support for the exchange, participants still described confronting habitual cultural standards that sometimes make alteration of practice patterns difficult. One respondent described the manner in which his efforts to improve his own relations with students were impaired by other faculty working with different (perhaps older) standards:

“With the students, it's nothing that I can do necessarily, because I become just a drop in the ocean. If they are free with me, then we rarely do rounds in solitude. Just as I am creating a rapport with them, my other friend or senior colleague walks in. They quickly build the barrier up again because the relationship with this other colleague is not the same as mine. “*Interviewee 4*

Another noted that while some students took readily to changes in teaching style that incorporated more journal article use and case-based learning, others were more hesitant because of their long experience in a lecture-based system. One interviewee discussed potential drawbacks to changes in the physician-resident relationship as well, voicing doubt as to whether residents at M-MakCHS were sufficiently self-motivated to continue to fulfill their responsibilities without what is currently the habitual threat of a more antagonistic relationship with their superiors.

Many interviewees noted changes in their clinical practice that have improved their physician-patient relationships. However, one respondent noted that while patients clearly respond to the sense of respect implicit in a patient-oriented approach to clinical care, many are not accustomed to, and therefore are hesitant to participate in, shared decision-making:

“The patients tell me that ‘Doctor, you make the decision. I'm just a layperson.’ And I'm trying to tell them we are now emphasizing patient-led care. I give you the information. You make the decision. But they are not forthcoming. It's a new concept. They don't know that they have that ability to make a decision.” *Interviewee 3*

### Effect of the Program on Brain Drain

Interviewees were asked to comment specifically on the role that the program may play in brain drain (i.e., the loss of human resources due to emigration or relocation, most often from less developed to more developed environments). All respondents agreed that the program has not led to brain drain, and most pointed to the fact that all participants have remained at Mulago, or elsewhere within the Ugandan health system. Half of interviewees cited MUYU’s focus on junior faculty (as opposed to younger trainees) as likely to play a role in preventing brain drain, as these are physicians who have generally established themselves within the system and within Uganda. Several respondents commented that the program may actually discourage brain drain to some extent; one interviewee was able to remain at M-MakCHS while awaiting faculty appointment only due to support provided by his involvement in a MUYU-related project, while another stated that the specialty skills provided to participants may make them more able to navigate and remain productive within the Ugandan medical system.

Although respondents denied that the program had led to brain drain, many voiced understanding of the complicated personal and systemic dynamics that can lead to brain drain. One interviewee commented:

“When you come back, and you've learned a lot, and you want to really be dynamic, and then you need something, and you write to the director, and it goes through procurement, and it spends two years—why not jump to another ship that is going to offer it within a few months and help you enhance your career in it? Potentially—I'm not saying it has led to brain drain—but potentially, you start to realize that if there is an area out there that is going to offer me a bit more than what I'm doing here, why not?” *Interviewee 4*

A few respondents suggested that the program could help to prevent brain drain further by providing more longitudinal support for its alumni, such as ongoing educational programming, or supporting other alumni projects in a way which would “make it attractive for the doctor to remain in Mulago” *(Interviewee 6)*.

### Lessons for Programmatic Improvement

Numerous possibilities for programmatic improvement were implicit in the participant interviews, and many were suggested explicitly. In addition to those discussed above (e.g., changes to financial support), suggestions included alterations to program scope and scale, improvements to mentoring structure, and refinements of pre- and post-participation programming.

#### Increase project scale and scope

Given that a number of the challenges to programmatic success are related to the relatively modest number of program participants operating in a larger health system and cultural context, it was common for interviewees to suggest expansion of the program. Interviewees suggested allowing more M-MakCHS faculty from internal medicine to participate, as well as expansion to other M-MakCHS faculty and staff. The most common suggestion was expansion to other physician groups, including pediatrics, OB/Gyn, neurology, radiology, and pathology. Several interviewees suggested including nursing in the exchange, as well as other clinical and support staff, including laboratory and radiology technicians, biotechnologists, and social workers. The inclusion of public health practitioners was suggested as well, specifically in relation to increasing the community impact of the program, and one respondent suggested the inclusion of clinical officers (a group of clinicians similar to Physician Assistants). Ultimately, these suggestions seemed to reflect a belief that programmatic participation was likely to benefit practitioners with various backgrounds, and that the health system as a whole was more likely to improve if efforts to build capacity were present across departments and cadres.

#### Improve programmatic mentoring systems

Discussions of the MUYU program mentoring systems revealed a nuanced situation in which some participants described productive relationships, while others cited sub-optimal mentorship as a barrier within the program and also as a major opportunity for improvement. All but one of the interviewees were at least "satisfied," and most were quite positive about their overall mentorship experiences. One respondent, however, described difficulty arranging specialty-specific mentorship (i.e., a mentor who practiced the same specialty), and another reported inconsistent quality of mentoring—with a very positive mentorship experience at one US hospital site but a poor experience at another.

Discussions supported the idea that a close, supportive mentor-mentee relationship could be vital for navigating difficulties, and ensuring achievement of educational goals during the exchange. Characteristics identified as important for a productive mentor-mentee relationship included a mentor with previous experience at M-MakCHS or another resource-poor environment; mentee involvement in mentor selection (ideally with the mentee having met the mentor prior to the exchange); a mentor who is specialty-specific; communication between the mentor and mentee to guide preparation prior to the mentee’s departure from Uganda; and a mentor who is present at, or can arrange appropriate supervision at, all clinical sites (if a single mentor who works at all sites is unavailable). As the pool of Yale faculty who have traveled to Uganda is limited, related suggestions were also made that the program arrange more regular visits by Yale faculty to M-MakCHS, both for teaching and to allow formation of mentor-mentee relationships.

#### Manage pre-departure expectations

Despite the difficulties that interviewees described related to financial issues, and limitations to hands-on experiences, the majority also stated that appropriate disclosure and discussion prior to participation made these issues easier to handle and would be unlikely to discourage participation among potential future participants. One respondent suggested that as part of this preparation, the program should support the pursuit of additional outside funding for all participants (such as Fulbright Scholarships, or funding from other medical specialty groups). Another interviewee suggested that perhaps some form of structured procedural documentation could allow participants further latitude in hands-on participation during their exchange. Establishment of specific educational objectives, or use of a pre-departure educational curriculum, ideally guided by a specialty-specific mentor, was also mentioned as likely to increase the educational achievements of participants. One interviewee commented on the selection process, and proposed that, to avoid imbalances as participants accrue in different areas of specialization, the selection of participants and their subspecialties should be made based on an assessment of need within the department and the hospital, rather than on participant preference.

#### Target resource support to ensure use of new capacity

While the MUYU program has been able to provide some durable medical equipment to returning program participants, such support has not been universally available. A majority of respondents suggested that the program focus further on the provision of medical resources specific to the new skills or capabilities of returning participants. Half of interview respondents also suggested increased support for research pursuits or the pursuit of research grants, and several participants also emphasized the importance of supporting an ongoing mentor-mentee relationship after participation, often as a means of supporting research and other projects.

#### Provide programming to ensure the maintenance of capacity

Suggestions for programming to ensure the maintenance of new capacity were common. Half of participants suggested arranging more regular visitation of Yale faculty to M-MakCHS. Most of these respondents recommended that faculty provide short courses on specific topics and make themselves available as future mentors, although some suggested that their visits be arranged to specifically support the specialty skills of returning MUYU participants, such as by providing post-exchange procedural training that participants were unable to garner in the US. Several respondents suggested that the program arrange CME activities for program alumni (independent of Yale faculty presence), and an equal number suggested that the program arrange semi-annual or annual alumni meetings to discuss and continue to amplify the effects of the program. A few participants suggested that return trips to the US were desirable or should be made available to program alumni, noting that these visits could be of shorter duration, as little time would be needed for reorientation prior to pursuing specific educational goals.

## Discussion

To our knowledge, this is the only study that has assessed the impact of training resource-poor partner faculty within the resource-rich partner environment. This study provides evidence that the MUYU Collaboration has had considerable beneficial effects on Mulago Hospital and Makerere University College of Health Sciences. The main effect of the program has been on the clinical and academic practice of exchange participants. Over the course of the program, the increasing number of MUYU participants has contributed directly to improved clinical patient care, and may have begun to encourage a significant shift in departmental attitudes towards issues like education and physician-patient relationships. Furthermore, the study supports the assertion that bidirectional educational exchanges in general have the potential to provide significant benefits to the resource-poor partner, despite historical concerns regarding the applicability of training, or the effects of such programs on brain drain, neither of which were supported by current findings.

Important lessons that may be drawn from both study findings and the accumulated experience of the MUYU Collaboration include:

- Although financial issues promise to be a perennial limitation of global health programming, a clear understanding of the burden that these experiences represent for participants, as well as solicitation of participants’ suggestions on novel solutions to these problems, will be vital to ensuring programmatic improvement.
- The importance of appropriate mentorship for exchange participants should not be underestimated, and may represent the most valuable resource not only for ensuring that educational goals are met, but also for managing the idiosyncratic and unexpected problems that are likely to arise in the course of such exchanges.
- While some challenges can be mitigated by appropriately managing expectations prior to departure, others, such as ensuring appropriate procedural experience, may require novel solutions such as special licensing and insurance permissions, or home-site mentoring following exchange participation.
- While the resource limitations of the resource-poor partner are unlikely to be solved via university-to-university collaborations, an appropriate focus on providing high-value durable medical equipment or other resources that are specific to newly acquired medical capacity is likely to be a high yield strategy.
- Collaborations should strive to provide post-participation support to alumni that will help them continue to apply and expand the capacity they have gained.
- Long-term commitment is likely to be required to ensure that the necessary critical mass is attained to effect improvements in the entrenched habitual practices and cultural standards of medical systems (including those of the resource-rich partner).

This study has several key limitations, including the small sample size. The number of respondents was limited by the MUYU participant pool and voluntary study participation. While the interviewees constituted the majority of MUYU participants, it is possible that participants who declined to be interviewed may have described different experiences within the program. Our respondent sample included only 1 woman, which is reflective of the gender disparity of participants overall, and of the junior faculty of the Department of Medicine at M-MakCHS, in which women remain a minority. While the responses of the female interviewee did not vary notably from her male counterparts, it remains possible that gender-specific differences exist in participant experience, and would require a larger sample size to detect. No consistent thematic differences were noted between the responses of other interviewee subgroups either (such as those who participated in the program as residents and those who participated as junior faculty), though the small sample size precludes in-depth subgroup analysis. Increasing sample size, however, would require either compulsory participation (which is both ethically problematic and unlikely to provide valid data), or inclusion of additional collaborations or exchange sites. Unfortunately, the scant number and modest scale of exchanges of this type, as well as their inherent variability across institutions, makes a multi-site study of this type infeasible.

The significant variation between global health programs at disparate institutions also limits the generalizability of study findings. The MUYU Collaboration and its programming have evolved to their current form through years of complex and dynamic institutional interactions. Not only does this make it difficult to isolate the effect of a single aspect of the MUYU Collaboration, but it may also limit the reproducibility of specific programming (and its benefits) outside of the context of the larger MUYU Collaboration. As such, the findings of this study cannot be generalized to non-MUYU programs, though they may serve as the basis for further investigation and innovation in the context of other collaborations.

Another limitation of this study is the risk of bias inherent in self-reported measures. Interview data rely upon subjective assessments to document programmatic effects (such as the self-perceived quality of teaching and patient care), rather than more standardized external assessments. Program effects may have been exaggerated if MUYU participants consistently overstated program effects on their behavior, had inaccurate perceptions of the effects of the exchange on their own behavior, or exaggerated the effect of their own work within the larger M-MakCHS system. Biased reporting of this type may be the result of misperception, or may relate to respondents’ desire to provide positive feedback to those conducting the study due to their presumed affiliation with the program (social desirability bias). The confidential nature of interview data, as well as the use of a trained Ugandan social scientist interviewer with no relationship to the program, were intended to reduce this bias, and are strengths of this study. Ultimately, while systematic error remains a possibility, the consistent claims of interviewees most likely point to a true effect, though one that remains difficult to quantify.

It should also be noted that a majority of respondents had participated in medical training in a resource-rich environment prior to the MUYU program. As an institution, M-MakCHS is relatively rich in international partnerships, and their faculty are relatively rich in prior international experiences. It is unclear whether a MUYU-type exchange would have similar effects in a more isolated environment. While interviewees were asked to describe only the effect of their MUYU experiences, it is impossible to determine the degree to which prior experiences may have contributed to perceived outcomes. Even if this contribution were considerable, however, the conclusion regarding the efficacy of resource-poor to resource-rich exchanges would remain the same.

Follow-up studies should aim to document program effects on more objective clinical outcomes, such as length of patient stay, complications of hospitalization, mortality, and perhaps even standardized measures of student or resident achievement, all of which may facilitate comparison to a control group or institution. Unfortunately, the complex nature of the Ugandan medical system, and its relatively limited infrastructure for data collection, make a study of this type impractical at this time.

Despite the above limitations, and the difficulties inherent to programming of this type, it is our hope that these findings will help support the creation of global health collaborations that are increasingly bidirectional, and therefore more likely to provide significant benefits to both partners [14, 15]. Our partners, after all, remain optimistic—one interviewee stated:

“The universe, and Uganda for that matter, is dynamic. Where we are now, we hope we shall be tenfold better in the future. But if we don't have this knowledge, then we'll be as complacent as thinking that where we are now is the best. I think the impact for me, as far as my knowledge was concerned, was that there is so much out there that we have been reading in textbooks, and not know that in actual practice, these blurbs as well as therapeutic modalities change lives. They save patients. We lose far too many patients because we don't have the diagnostic and therapeutic capability. But I am full of hope that the future is bright for us.” *Interviewee 4*

## Supporting Information

S1 Text Qualitative Interview Protocol of M-MakCHS Faculty MUYU Participants. (DOCX)
