# Supplementary material for: The Effect of a Bidirectional Exchange on Faculty and Institutional Development in a Global Health Collaboration
Source: PLoS One. 2015 Mar 23;10(3):e0119798. doi: 10.1371/journal.pone.0119798 (PMC4370667; doi:10.1371/journal.pone.0119798)
Supplement: S1 Text — (DOCX) [file pone.0119798.s001.docx]

**Text S1. Qualitative Interview Protocol of M-MakCHS Faculty MUYU Participants**

**Structure of the interview:**

1. General questions
2. Questions regarding the impact of training at Yale on knowledge, skills and professional development
3. Questions regarding impact on attitude and professionalism
4. Questions regarding impact on the institution and community
5. Questions regarding capacity-building
6. Final comments
7. **General questions:**
   1. Please tell me about why you were interested in spending time at Yale.
      1. Probe question: What were your top three reasons for wanting to go to Yale?
   2. Please tell me about your pre-departure preparation.
      1. How did you prepare for your time at Yale?
      2. How well prepared were you for your experience at Yale?
      3. How could your pre-departure preparation have been done better?
   3. Please tell me about your initial experience upon arriving in the US.
      1. How did your colleagues at Yale receive you?
      2. Did you feel welcomed by the other doctors at Yale? Why, or why not?
   4. Please tell me about your orientation at Yale.
      1. Was the role you were assigned once you were working at the hospital in line with your expectations? If yes, tell me about it. If no, why not?
   5. Please tell me about your supervision and mentorship at Yale.
      1. Did you have a primary mentor (someone who felt responsible for your training)? If so, how often did you meet?
      2. How comfortable did you feel with your mentor?
         1. Were you able to go to her/him if you had any problems – professional or personal? Why or why not?
      3. What is your relationship like with your Yale mentor now that you have returned to Uganda? Are you still in contact? How comfortable would you feel contacting him or her to ask for advice at this point?
   6. Please tell me about your housing and meals while at Yale.
      1. Did you feel they were adequate? Why? What suggestions do you have for improvement?
   7. Please tell me about your financial compensation and stipend.
      1. How adequate was your stipend? Were there things you not able to afford that you would have liked to?
      2. How did this affect your family and dependents in Uganda?
      3. Do you have any suggestions for improvement?
8. **Questions regarding the impact of training at Yale on knowledge, skills and professional development**
   1. Did your experience at Yale impact your fund of medical knowledge? If so, how?
      1. What specific area of practice did you focus on at Yale?
      2. Has the experience or medical knowledge you gained at Yale impacted your clinical practice in Uganda? If so, please provide specific examples. If not, why not?
   2. Please tell me about any specific medical skills, such as procedures or techniques, that you learned at Yale.
      1. Were you able to acquire the skills that you were planning to learn?
      2. Was there knowledge or skills that you hoped to learn at Yale but were unable to? If so, what? And why were you unable to learn them?
      3. Was there knowledge or skills that you learned that you did not expect?
      4. Are you now using the skills that you learned at Yale in Uganda? If so, how? If not, why not?
   3. How has the experience at Yale impacted on your role and practice:
      1. As a clinician?
      2. As a teacher?
      3. As a researcher?
      4. On your career in general
   4. Has the training at Yale impacted your leadership role at Mulago/Makerere? Please explain.
9. **Questions regarding impact on attitude and professionalism:**
   1. The way medical professionals interact with colleagues and other medical staff often differs between countries and cultures. Please compare and contrast the relationships between the following groups at Yale versus at Makerere:
      1. Medical faculty and students
      2. Medical faculty and residents
      3. Medical faculty and colleagues
      4. Physicians and nurses
   2. Has your relationship with any of the above groups (i.e., students/residents/colleagues/nurses) changed because of your experience at Yale? In what way?
   3. Has your relationship with your patients and their families changed because of this experience? In what way?
10. **Questions regarding impact of MUYU on the institution and community:**
    1. What do you think is the impact on Mulago Hospital of Yale medical staff (faculty, residents, students) coming to work at Mulago?
    2. What do you think is the impact on Mulago Hospital of Mak CHS medical staff (faculty, students) training at Yale and then coming back to Mulago?
    3. Has the MUYU Collaboration (i.e., both of the groups above) impacted patient care at Mulago? If so, in what way? If not, why not?
    4. Has this collaboration impacted undergraduate medical training? If so, in what way? If not, why not?
    5. Has this collaboration impacted post-graduate SHO training? If so, in what way? If not, why not?
    6. Has this collaboration impacted research at Mulago? If so, in what way? If not, why not?
    7. Has this collaboration impacted the community that is served by Mulago in any way? If so, in what way? If not, why not?
       1. Probe: What about new interventions designed to go into the community, such as the MammoVan?
       2. Should the project focus more, or less on these community interventions? Why?
11. **Questions regarding return to Uganda**
    1. Were there any challenges finding employment or returning to your regular duties when you returned to Uganda?
    2. How was your experience abroad perceived by your colleagues and administration?
    3. What were the sacrifices that you made in order to go to Yale?
       1. Probe: Were there financial consequences?
       2. Or consequences related to being away from your family?
12. **Questions regarding capacity-building**
    1. How would you define capacity building?
    2. A major goal of the MUYU project is to help build Mulago’s capacity to train the Ugandan physician workforce by furthering the training of Mulago’s teaching staff. Do you think the MUYU Collaboration has helped to build this capacity at Mulago and/or Makerere? If so, in what ways has it accomplished this? If not why not?
    3. What factors have impeded capacity building at Mulago and/or Makerere?
    4. What capacity do you think is most lacking at Mulago/Makerere? Should MUYU consider focusing on other types of capacity building as well? Why?
    5. What effect do you think the MUYU program has on “brain drain”, i.e. Ugandan physicians leaving Uganda in order to practice medicine outside of Uganda? Do programs like this encourage or discourage brain drain?
       1. If so, do you consider this a problem with the program?
       2. What, if anything, should the program do to change its effect on brain drain?
13. **Final Comments**
    1. Would you like to make any other comments about any aspects of MUYU (as it has impacted you, your colleagues, the institution and the community)?
    2. What other suggestions, if any, do you have for improvement to the program?
    3. Do you have any other final comments?
